# Identifying the Most Effective Recruitment Strategy Using Financial Reimbursements for a Web-Based Peer Network Study With Young People Aged 16-18 Years: Protocol for a Randomized Controlled Trial

**DOI:** 10.2196/44813

**Published:** 2023-08-11

**Authors:** Michelle Raggatt, Cassandra J C Wright, Rachel Sacks-Davis, Paul M Dietze, Margaret E Hellard, Jane S Hocking, Megan S C Lim

**Affiliations:** 1 Burnet Institute Melbourne Australia; 2 School of Public Health and Preventative Medicine Monash University Melbourne Australia; 3 Menzies School of Health Research Darwin Australia; 4 Melbourne School of Population and Global Health University of Melbourne Parkville Australia; 5 National Drug Research Institute Curtin University Melbourne Australia; 6 Department of Infectious Diseases Alfred Health Melbourne Australia

**Keywords:** young adult, incentive reimbursement, research subject, study participant, financial, research subject recruitment, social network, peer network, web-based network, randomized, friend, recruit, incentive, reimburse, reward, incentivized, youth, adolescent, teenage, recruitment, reinforcing factor, enabling factor, disambiguation, intrinsic incentive, extrinsic incentive, motivation, reward system, positive reinforcement, compensation, monetary, remuneration, remunerative incentive, financial incentive, bonus, stipend, donation

## Abstract

**Background:**

Peers are an important determinant of health and well-being during late adolescence; however, there is limited quantitative research examining peer influence. Previous peer network research with adolescents faced methodological limitations and difficulties recruiting young people.

**Objective:**

This study aims to determine whether a web-based peer network survey is effective at recruiting adolescent peer networks by comparing 2 strategies for reimbursement.

**Methods:**

This study will use a 2-group randomized trial design to test the effectiveness of reimbursements for peer referral in a web-based cross-sectional peer network survey. Young people aged 16-18 years recruited through Instagram, Snapchat, and a survey panel will be randomized to receive either scaled group reimbursement (the experimental group) or fixed individual reimbursement (the control group). All participants will receive a reimbursement of Aus $5 (US $3.70) for their own survey completion. In the experimental group (scaled group reimbursement), all participants within a peer network will receive an additional Aus $5 (US $3.70) voucher for each referred participant who completes the study, up to a maximum total value of Aus $30 (US $22.20) per participant. In the control group (fixed individual reimbursement), participants will only be reimbursed for their own survey completion. Participants’ peer networks are assessed during the survey by asking about their close friends. A unique survey link will be generated to share with the participant’s nominated friends for the recruitment of secondary participants. Outcomes are the proportion of a participant’s peer network and the number of referred peers who complete the survey. The required sample size is 306 primary participants. Using a multilevel logistic regression model, we will assess the effect of the reimbursement intervention on the proportion of primary participants’ close friends who complete the survey. The secondary aim is to determine participant characteristics that are associated with successfully recruiting close friends. Young people aged 16-18 years were involved in the development of the study design through focus groups and interviews (n=26).

**Results:**

Participant recruitment commenced in 2022.

**Conclusions:**

A longitudinal web-based social network study could provide important data on how social networks and their influence change over time. This trial aims to determine whether scaled group reimbursement can increase the number of peers referred. The outcomes of this trial will improve the recruitment of young people to web-based network studies of sensitive health issues.

**International Registered Report Identifier (IRRID):**

DERR1-10.2196/44813

## Introduction

### Background

Late adolescence is an important time in health and social development when many potentially modifiable health risks arise. This period often includes the exploration and initiation of behaviors including sexual activity, alcohol and other drug use, and the emergence of mental health problems [1]. Peer relationships are one of many interrelated determinants of health during adolescence [1]. For instance, adolescents with peers who engage in tobacco, alcohol, and cannabis use are more likely to initiate these behaviors themselves [2,3]. Despite the acceptance of peers as an important determinant of health, there is little recent quantitative research examining the influence of adolescents’ peer networks compared to research focused on the individual. Furthermore, the social environment that adolescents live in is constantly evolving, with more interactions taking place on the internet than in previous generations [4]. There is therefore a need for updated research about the influence of peers in the current context that leverages these new interactions and social connections.

Social network theory is an approach used to understand the influence of adolescents’ peers, among other things [5]. In the context of adolescent health behaviors, social network research involves the collection and analysis of information about relationships between individuals and their health behaviors [6]. Research seeks to understand the influence of a peer network and the mechanisms of this influence (eg, social pressure, communication of attitudes and norms, and social support) [6]. Understanding peer influence and its mechanisms can be used to design health interventions that leverage the strengths of adolescents’ social networks [7]. The majority of prior social network studies on adolescents have recruited participants from schools, with the “network” cast as entire classes or schools [3]. This recruitment setting produces high response rates and high ascertainment of contacts and links. It also has the potential for demographic diversity within the sample (particularly if several different schools were selected for the study). However, many longitudinal studies of this type were designed with relatively short follow-up periods (eg, 1-2 years) due to students changing classes and leaving school [8-10]. Studying a whole network (eg, an entire school or class) also limits the generalizability of findings to the wider adolescent population. As the network boundary is restricted, respondents are commonly limited to nominating friends from a directory within their class or their school year [11,12]. Alternative recruitment settings in social network research are community or health organizations. These settings were used by studies that sought specific subgroups (eg, youth experiencing homelessness or those with a specific health condition) and where recruitment staff had pre-established relationships with the participants [13,14]. However, studies recruiting from these settings reported difficulties reaching the desired sample sizes at sufficient speed [15,16]. Moreover, analyses of peer influence and sociometry in these studies were limited to egocentric data, meaning that primary participants report on the characteristics and behaviors of their friends, which limits the accuracy and reliability of behavioral reports [14].

Conducting social network research on peer groups using web-based techniques could overcome some of these disadvantages and has the potential to be used in longitudinal studies for the ongoing collection of information about people’s social networks beyond school. More broadly, web-based mechanisms are increasingly being used to recruit young people, as researchers have found web-based studies can effectively reach many young people, including those in more marginalized groups such as young people who identify as lesbian, gay, bisexual, transgender, queer, asexual, or other (LGBTQA+) [17,18]. Web-based questionnaires also provide greater privacy during completion than in person- or school-administered questionnaires, which is particularly important for sensitive topics such as sexual health and substance use [19]. To our knowledge, there are no social network studies with adolescents conducted entirely on the internet, but web-based cross-sectional, individual-level surveys have used social media recruitment, specifically Facebook, to recruit young people [17,20,21]. Social media platforms rapidly emerge, update, and outdate, resulting in frequent changes to their popularity and hence their usefulness as recruitment sites.

Web-based respondent-driven sampling (webRDS) offers a model for how we could recruit adolescents’ peer networks. Like traditional respondent-driven sampling (RDS), webRDS involves primary participants who are incentivized to recruit a specific number of peers into the study by offering a monetary reimbursement per recruited peer [22]. Studies using webRDS have either recruited the primary participants in person through study administrators [22,23] or used targeted Facebook advertising and phone screening to enroll primary participants [24]. Referral of secondary participants has been facilitated using emails [22,24] or private messages primarily via Facebook [23,24]. Reported benefits of using webRDS were that it was quicker, cheaper, and overcame barriers related to participants distributing physical coupons compared to RDS [23]. Potential limitations and considerations of webRDS include differences in participant demographics and risk profiles between recruitment using RDS and webRDS [23]. Fraudulent or duplicate cases were identified as a problem due to some participants trying to increase their reimbursement by creating false email addresses to artificially increase their number of referrals [24]. The reimbursements offered for survey completion and friend referral varied between each study, and the impact of the reimbursement was not tested. Therefore, the extent to which a reimbursement affects the recruitment of peers remains an important practical question that impacts the cost and feasibility of conducting a web-based social network study.

### Rationale

Our study will address gaps identified in previous research by determining whether a web-based approach is effective in recruiting adolescent peer networks. We will also address how different reimbursement models impact recruitment. In group 1 (experimental group: scaled group reimbursement), all participants within a peer network will receive an additional Aus $5 (US $3.70) voucher for each referred participant who completes the study, up to a maximum total value of Aus $30 (US $22.20) per participant. In group 2 (control group: fixed individual reimbursement), participants will only be reimbursed for their own survey completion.

If a web-based social network study proves feasible, this method has the potential to overcome barriers related to conducting longitudinal network research and use this web-based approach to better examine how social networks and their influence change over time. Identifying the characteristics associated with successful peer network recruitment would enable us to determine whether adolescents with certain key demographics or at-risk groups are more or less likely to refer their peers. By identifying these groups, we can gather further feedback and tailor the study methods in the future so that these social networks may be better captured.

### Aims and Research Questions

The primary aim of this study is to compare the effectiveness of 2 different financial reimbursement strategies for recruiting young people into a web-based peer network study. This will be explored through the following research questions:

1. Does a scaled group financial reimbursement for peer referral increase the proportion of an individual’s close friend network that completes the survey compared to having no peer referral reimbursement?
2. Does a scaled group financial reimbursement for peer referral increase the number of close friends per participant who complete the survey compared to having no peer referral reimbursement?

We hypothesize that a scaled group financial reimbursement for peer referral will result in a significantly higher proportion of participants’ close friend networks and a significantly higher number of close friends per participant who complete the survey, compared to a fixed reimbursement rate for individuals.

The secondary aim is to determine what demographic and behavioral factors are associated with successfully recruiting close friends.

## Methods

### Study Design

**Figure 1:**
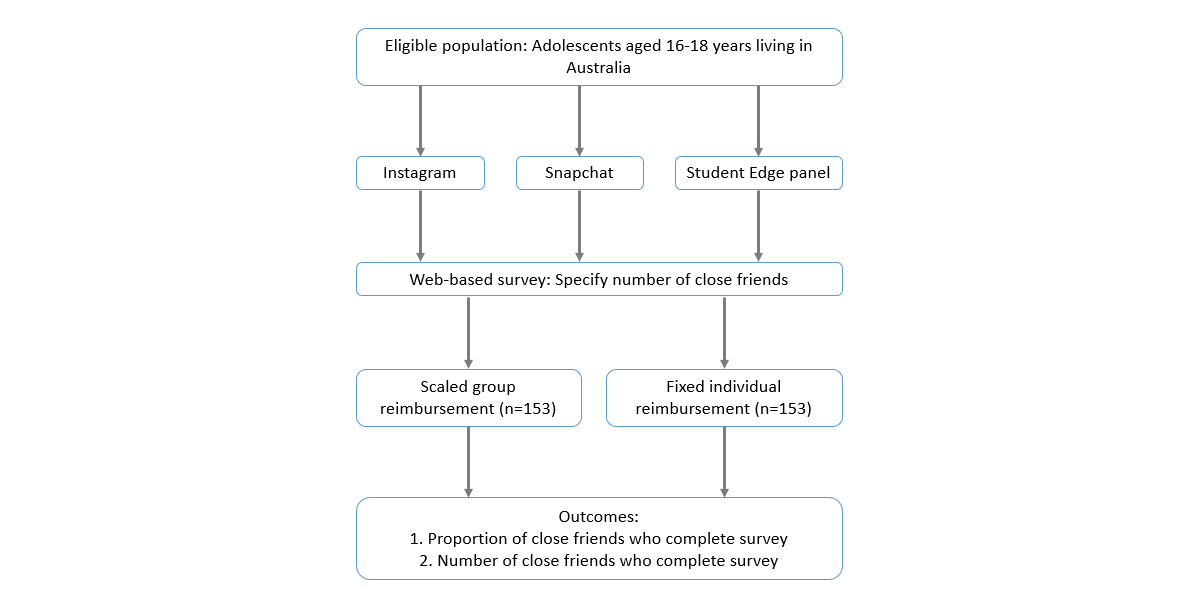
Study design.

This study uses a 2-group randomized trial design to test the effectiveness of reimbursements for peer referral in a web-based cross-sectional peer network survey. Participants will either be randomized to scaled group reimbursement (the experimental group) or fixed individual reimbursement groups (the control group). The trial will be of 60 days duration. Figure 1 illustrates the study design.

### Participants and Eligibility

This trial involves both primary participants and secondary participants. Primary participants are defined as individuals recruited through the recruitment platforms (Instagram, Snapchat, and the Student Edge panel) and are able to refer their close friends. Secondary participants are individuals nominated by a close friend and referred to the study by a primary participant.

Primary participants will be eligible if they are aged between16 and 18 years and are currently living in Australia. Secondary participants will be eligible if they are aged 16 years or older and currently living in Australia.

### Intervention

All primary and secondary participants will receive Aus $5 (US $3.70) as reimbursement for their own survey completion. Primary participants will be randomized to either (1) scaled group reimbursement (experimental) or (2) fixed individual reimbursement (control). In the scaled group reimbursement, all participants within a peer network (ie, the primary participant and all secondary participants) will receive an additional Aus $5 (US $3.70) voucher for each added secondary participant that the primary participant recruits to the study, up to a maximum total value of Aus $30 (US $22.20) per participant. The reimbursement per participant stops increasing at 6 per peer group, but up to 11 people in a peer group can be referred and reimbursed up to Aus $30 (US $22.20) each (1 primary and 10 secondary). In the fixed individual reimbursement, all participants will only be reimbursed Aus $5 (US $3.70) for their own survey completion. Table 1 describes the reimbursement amounts for participants in each group depending on the number of secondary participants who complete the survey within their friendship group.

**Table 1:** Reimbursements for each intervention group.

Number of participants in friend group who complete survey	Reimbursement value per participant	
	Scaled group reimbursement (experimental)	Fixed individual reimbursement (control)
1 primary; 0 secondary	Aus $5 (US $3.70)	Aus $5 (US $3.70)
1 primary; 1 secondary	Aus $10 (US $7.40)	Aus $5 (US $3.70)
1 primary; 2 secondary	Aus $15 (US $11.10)	Aus $5 (US $3.70)
1 primary; 3 secondary	Aus $20 (US $14.80)	Aus $5 (US $3.70)
1 primary; 4 secondary	Aus $25 (US $18.50)	Aus $5 (US $3.70)
1 primary; 5-10 secondary	Aus $30 (US $22.20)	Aus $5 (US $3.70)

Reimbursements are in the form of a gift voucher sent by SMS to participants’ mobile numbers using a third-party service provider at the end of the trial period. However, due to the procedures of Student Edge, primary participants recruited through this setting will receive the initial Aus $5 (US $3.70) reimbursement on survey completion through Student Edge and further reimbursements via SMS at the end of the study, if applicable.

### Outcomes

The primary outcome is defined as the proportion of primary participants’ close friends who complete the survey. Survey completion is recorded if the secondary participant clicks the final survey submission button. This outcome measure indicates the level of complete data about each primary participant’s close friend network.

The secondary outcome is the number of close friends who complete the survey per primary participant. This outcome measure assesses whether the reimbursement model results in an overall higher number of participants.

### Sample Size

We calculated the required sample size to test the difference in proportion of an individual’s close friend network that completes the survey between the intervention and control groups. Based on previous literature, we estimated that the average number of close friends listed in the survey (nominations) is expected to be 3 [9,11,25], and that approximately half of the primary participants in the scaled reimbursement group would successfully recruit at least one friend into the study [22,23]. Based on these data and assuming that the probability of recruiting each friend is binomial, with the probability of recruiting no friends being (1–*p*)^*n*, where *n* is the total number of friends and *p* is the probability of each friend being recruited, we estimated that the probability of any one nomination being recruited would be 0.2. We assessed the sample size required for 80% power to detect a 2-fold difference in proportion between the scaled group reimbursement and fixed individual reimbursement groups. We assumed some variation between friendship groups and considered each friendship group as a cluster, with an assumed intracluster correlation (ICC) of 0.5 and a mean cluster size of 3, with a coefficient of variation for cluster size of 0.83. Based on these assumptions, 153 primary participants must be randomized to each group, and a total sample of 306 primary participants is required. Based on this sample size, we will also be able to detect a difference in the number of close friends per participant who complete the survey of 1.4 and 80% power. In total, across both groups, we estimate that at least 92 secondary participants will be recruited based on conservative assumptions.

Since we plan to recruit through 3 primary participant recruitment settings, we intend to recruit 102 primary participants from each setting (Instagram, Snapchat, and the Student Edge panel). Recruitment in each setting will continue until 102 primary participants are reached, or 6 weeks have passed. If fewer than 102 participants are recruited from any setting, further recruitment will be conducted from successful settings.

### Recruitment

Primary participants will be recruited from 3 web-based platforms: Instagram advertising, Snapchat advertising, and the Student Edge panel. Specific details about each recruitment platform and how participants will be recruited are outlined below:

- Instagram is a free-to-use photo and video sharing social network service predominantly used through smart phone apps. Instagram allows users to engage with a range of advertisements outside of their immediate social network. Instagram is currently owned by Meta, which administers Instagram’s advertising manager function. Using the advertising manager, businesses and users can manage paid advertisements on Instagram. The study will use the advertising manager to select target ages 16-18 years and limit the geographic area to Australia. The advertisement will feature images relating to the survey topics and text outlining the study. Tapping or swiping on the Instagram advertisement will direct prospective participants to the study information page.
- Snapchat is an instant messaging app that allows users to share pictures and videos (called “snaps”). Messages are usually only available to recipients for a short period before they can no longer be viewed. The study will use Snapchat’s paid advertising feature to select the target audience of people aged 16-18 years, within the geographic area of Australia. Paid advertisements in the form of a short video appear between viewing friends’ snaps, and people can swipe up to interact with the ad. The advertisement will feature a short, animated video with text outlining the study. By tapping on the advertisement, prospective participants will be redirected to the study information page.
- Student Edge [26] is a web-based organization targeted toward people in high school, technical and further education (TAFE), and university in Australia. The website provides students with study tips, news, promotions, competitions, job listings, and surveys. People can voluntarily sign up for the Student Edge Surveys panel to earn rewards. Student Edge will send an email invitation to prospective participants aged 16-18 years, who have signed up for their panel. Participants then click on the link in the email and are redirected to the study survey page.

Secondary participants are recruited by primary participants. When primary participants reach the end of the survey, a default message and URL unique to them and their friendship group will be generated. The primary participant is asked to discuss the project with their friends and share the message and URL. Secondary participants can click on the link to take them to the study information page and then proceed to the survey. One reminder email is sent to the primary participant 72 hours after they complete the survey. Secondary participants are not given the option to refer additional friends (ie, there is only 1 wave of secondary recruitment).

All participants will be required to provide their email address and mobile phone number. To prevent multiple responses from the same individual, repeat email addresses and mobile phone numbers will be detected by Research Electronic Data Capture (REDCap; Vanderbilt University), which is used to collect data (see below), and these participants will not be able to proceed with the survey.

### Allocation of Intervention

Primary participants are randomized upon opening the study webpage. Randomization is managed through a REDCap survey form to enable automatic allocation independent of the researchers. The timestamp when a participant opens the webpage determines their allocated group, with those opening at an even number of seconds assigned to the scaled group (intervention) and at an odd number of seconds to the fixed group (control). Primary participants are informed about their intervention group after they answer how many close friends they have. Secondary participants are assigned to the same intervention group as the primary participant who recruited them. Researchers will not be blinded to the intervention group as they have no direct contact with the participants.

### Data Collection

Participants will complete a web-based survey using the REDCap data collection tool [27]. The survey will capture information about peer networks, mental health, alcohol and other drug use, COVID-19 vaccination, sexual health, social media, and media related to sexual health, including pornography, sexting, and dating apps. The survey begins with a study information webpage containing a brief video accompanied by written information explaining the purpose of the research, what is involved, and how to participate. After the study information, participants proceed to a sign-up or consent page. Participants provide their contact details (email and mobile number) and will be asked to complete a checkbox list indicating their informed consent to confirm they understand and agree to provide their information and the use of that information for the purpose of the study. Participants complete a 2-step authentication by entering a code sent to their nominated mobile number. Participants will then be directed to complete the survey. If the individual is a primary participant, a unique URL will be generated at the end of the survey to share with their friends.

The target peer networks for recruitment are assessed by asking primary participants about their close friends. Participants are asked how many people they consider to be their close friends and to give their initials or a codename for each. Participants proceed to answer questions about each friend’s age, gender, and perceived closeness. If participants have more than 10 close friends, they are asked to answer the questions for their 10 closest friends. Therefore, the possible range of nominated close friends who may be referred to as secondary participants is 0 to 10. A network size of up to 10 was deemed sufficient, as in other studies, most young people nominate only 2-3 close friends [9,27]. A limit was necessary to minimize the burden on participants to answer too many questions and manage the study’s budget for participant reimbursements.

Potential factors associated with close friends completing the survey are collected through the web-based survey. Primary participant-level factors include primary participant demographic characteristics, recruitment settings (Instagram, Snapchat, and the Student Edge panel), and health outcomes and behaviors. Health domains include mental health and well-being, sexual health, social media use, technology use related to sex, alcohol, tobacco smoking, other drugs, and COVID-19 vaccination. Friend-level predictors include network size and close friends’ gender, age, perceived closeness, and frequency of discussing various health-related topics.

### Data Management

Survey data for participants within the same peer network group will be linked using the unique auto-generated survey URL. Secondary participants’ survey data will be linked to the primary participants’ reported data using codenames and initials.

### Statistical Analysis

All analysis will be conducted in Stata (version 15; StataCorp). We will first compare the 2 baseline groups to determine whether they are comparable on key demographic factors. If not, further analysis will be adjusted for any differences.

Our primary analysis will assess the effect of the reimbursement intervention on the proportion of primary participants’ close friends who complete the survey using a multilevel logistic regression model. In this model, close friend nominees are the lowest level of data and are grouped by primary participant. Each close friend nominee can either be successfully recruited or not recruited (a binary outcome).

For aim 2, to determine the effect of the intervention on the number of close friends per primary participant who complete the survey, we will use a regression model with a count distribution appropriate for the data.

In secondary analysis, we will identify factors associated with successfully recruiting each close friend by including friend-, relationship-, and primary participant–level variables. We will use a logistic regression model where the outcome is whether a primary participant successfully recruited at least one friend or recruited zero friends, and the potential predictors include recruitment setting, demographics, health variables, and network size.

### Data Monitoring

This study is the first time we have tested this referral approach. The number of reimbursements needed depends on the number of secondary participants. Therefore, we plan to monitor the secondary participant referral rate during data collection to ensure we do not exceed the budget for reimbursements (Aus $20,000; US $14,800). If the referral rate indicates we will exceed this budget, we will stop advertising the project and close the survey early to new primary participants. We will monitor the secondary participant referral rate at recruitment of 50, 100, 150, and 200 primary participants.

### Community Engagement

Young people were involved in the development of the study design through previous focus groups and interviews [28]. Twenty-six participants aged between 16 and 18 years living in Victoria were recruited through paid advertisements on Facebook and Instagram. In these interviews, young people were asked about their perspectives on acceptable and engaging ways to conduct peer network research. Young people identified recruitment settings and strategies to communicate the study to participants. For the recruitment of social networks, young people endorsed having the autonomy to ask their friends if they would like to participate and using a referral link. Young people recommended using reimbursements to overcome barriers to participation and to show them that the researchers value their participation and experience. Young people suggested including video, more information about the researchers conducting the project, and a visually appealing design for research materials to improve how research participation is communicated to them, build trust with the research organization and increase their engagement in the research project. The researchers developed the research questions and outcomes for this study. Young people then gave their feedback on the acceptability of the survey topics and suggestions to improve their comfort with answering sensitive questions. This study is informed by this previous work and will test the strategies identified by young people. Young people who participated in formative development will not be involved in the conduct of this trial.

### Ethics Approval

This study has been approved by the Monash University Human Research Ethics Committee (23132). Our study involves primary participants aged 16-17 years, and some secondary participants will also be aged 16-17 years. Individuals aged 16-17 years can have the competence and capacity to give informed consent without parental knowledge or permission [29]. As this study takes place on the internet and aims to ask participants about sensitive topics such as pornography and sexual health, it would be inappropriate and uncomfortable to require parental consent for participation in the study. If participants wish, they are encouraged to discuss the study with their parents or guardians. Despite being a randomized controlled trial, the study was not registered, as the primary outcome (recruitment of friends) was not a direct health or medical outcome.

Some primary participants may be concerned about their friends’ privacy, as they are sharing information about their friends. We will only collect the minimal required information about friends to enable linking data within peer networks. We are not collecting identifiable information from primary participants about their friends (such as friends’ email addresses or mobile numbers). Friends choose to voluntarily participate and provide their own contact information directly.

To further protect friends’ right to freely choose to participate, primary participants will not be informed about whether their friend has signed up for the study. A reminder email contains a generic message and disclaimer to ignore it if they have already discussed the project with their friend.

We will disseminate our findings through peer-reviewed publications, conference presentations, and a PhD thesis. A summary of results will be sent to participants and posted on the research organization’s website.

## Results

Participant recruitment commenced in March 2022 and was completed in May 2023. Data analysis has not yet commenced.

## Discussion

### Principal Findings

This new social network research study about young people’s peer networks uses web-based methods (ie, recruitment, peer referral, and survey). If feasible and effective, our study design could be used to overcome previous barriers to conducting longitudinal social network research with young people and take advantage of the benefits of using web-based methods (eg, reach marginalized groups and improve privacy). The randomized controlled trial design will enable us to determine the effect of 2 different reimbursement models on the recruitment of young people’s peer networks. The limitations of this study are convenience sampling and cross-sectional design, which limit the extent to which the findings would apply to a longitudinal study.

## Abbreviations

ICC: intracluster correlation
LGBTQA+: lesbian, gay, bisexual, transgender, queer, asexual, or other
TAFE: technical and further education
RDS: respondent-driven sampling
webRDS: web-based respondent-driven sampling
